# Radiomics-Driven Tumor Prognosis Prediction Across Imaging Modalities: Advances in Sampling, Feature Selection, and Multi-Omics Integration

**DOI:** 10.3390/cancers17193121

**Published:** 2025-09-25

**Authors:** Mohan Huang, Helen K. W. Law, Shing Yau Tam

**Affiliations:** 1 School of Medical and Health Sciences, Tung Wah College, Hong Kong SAR, China; mhhuang@twc.edu.hk; 2 Department of Health Technology and Informatics, The Hong Kong Polytechnic University, Hong Kong SAR, China

**Keywords:** clinical translation, feature selection, imaging modality, multi-omics, prognosis prediction, radiomics, sampling methods, tumor prognosis

## Abstract

Radiomics has shown remarkable potential in predicting cancer prognosis by noninvasive and quantitative analysis of tumors through medical imaging. This review summarizes recent advances in the use of radiomics across various cancer types and imaging modalities, including computed tomography (CT), magnetic resonance imaging (MRI), ultrasound, positron emission tomography (PET), and interventional radiology. Innovative sampling methods, including deep learning-based segmentation, multiregional analysis, and adaptive region of interest (ROI) methods, have contributed to improved model performance. The review examines various feature selection approaches, including least absolute shrinkage and selection operator (LASSO), minimum redundancy maximum relevance (mRMR), and ensemble methods, highlighting their roles in enhancing model robustness. The integration of radiomics with multi-omics data has further boosted predictive accuracy and enriched biological interpretability. Despite these advancements, challenges remain in terms of reproducibility, workflow standardization, clinical validation and acceptance. Future research should prioritize multicenter collaborations, methodological coordination, and clinical translation to fully unlock the prognostic potential of radiomics in oncology.

## Simple Summary

This review explores how radiomics, a method using medical imaging such as computed tomography (CT) and magnetic resonance imaging (MRI) scans to analyze tumors in detail, improves cancer prediction. Radiomics turns images into measurable data and helps researchers to understand tumor behavior. New techniques, such as using deep learning methods to outline tumors and investigate at different areas within them, are making radiomics more consistent and accurate. This review examines ways to select the most useful information from various imaging modalities and combine this with other patient information to better predict cancer outcomes. While radiomics holds great promise, challenges remain in making sure that the results are consistent across different hospitals and that the methods used are standardized and accepted by the clinician communities. Future research should focus on larger studies, better coordination of methods, and practical use in clinics to fully unlock radiomics’ potential for improving cancer care.

## 1. Introduction

Radiomics is a rapidly developing field in oncology that extracts quantitative and reproducible information from clinical images to reveal complex patterns and hidden information that elude human visual perception [1,2]. It is a non-invasive method that transforms medical images into high-dimensional data through high-throughput analysis [2]. By extracting and analyzing quantitative radiomic features (such as intensity, shape, texture, and wavelet-based patterns) from imaging modalities like computed tomography (CT), magnetic resonance imaging (MRI), or positron emission tomography (PET), radiomics identifies imaging biomarkers that are linked to tumor biology and clinical outcomes [3,4,5]. Recently, modalities including ultrasound, radionuclide imaging (e.g., single-photon emission computed tomography), and interventional radiology have been included in radiomics research. These modalities have shown potential in tumor characterization, treatment monitoring, and prognosis prediction [6,7,8]. These features provide valuable information about tumor phenotypes and the tumor microenvironment, which can support clinical decision-making [9].

Radiomics increasingly contributes to prognosis prediction across various cancers, providing robust models for endpoints such as overall survival, progression-free survival, recurrence, and treatment response [10]. Significant prognostic value has been observed in non-small-cell lung cancer, glioblastoma, breast cancer, and hepatocellular carcinoma. Studies have shown that radiomic feature profiles can offer additional risk stratification after treatment, such as evaluating a patient’s suitability for immunotherapy based on tumor microenvironment characteristics. This ability to decode tumor heterogeneity and phenotypic characteristics helps to support personalized treatment planning and clinical decision-making, ultimately contributing to more precise and individualized cancer care across oncological disciplines.

Despite advances integrating radiomics with multi-omics data and precision medicine, challenges remain in clinical translation. The most important challenges identified are data heterogeneity and lack of standardization [11]. Differences in imaging protocols, scanner manufacturers, acquisition parameters, and reconstruction algorithms across institutions can lead to significant variations in radiomic feature values, undermining their reproducibility and generalizability. Manual or semi-automated tumor segmentation introduces inter-observer variability, complicating workflow consistency. Moreover, high-dimensional, sparse radiomics datasets increase overfitting risk, threatening model robustness [12].

In the specific workflow of radiomics, sampling methods and feature selection are promising approaches to address the above issues. They are key steps in building stable, interpretable, and high-performing models and are the focus of most current studies [13]. For example, the sampling method used to extract two-dimensional or three-dimensional regions of interest (ROIs) from images is closely related to the goal of the radiomics study [14]. Feature selection is performed through algorithms to find the best subset of features that are most relevant to the research objective [15]. In recent years, researchers have developed and evaluated various sampling algorithms, including filtering, wrapper, and embedded methods [13]. More recently, hybrid techniques, ensemble learning, and multi-omics integration have further expanded the capabilities of radiomics-based prognosis models [16].

This review comprehensively explores radiomics sampling and feature selection applications in tumor prognosis prediction, critically assessing methodological advances in segmentation, feature extraction and selection to enhance model reliability and accuracy. It compares imaging modalities (CT, MRI, PET, ultrasound) regarding their prognostic contributions and evaluates emerging deep learning techniques shaping radiomics’ future. The review also addresses challenges to clinical implementation such as standardization, reproducibility, interpretability, and regulatory hurdles, concluding in recommendations for future research to overcome barriers and accelerate clinical adoption of radiomics.

## 2. Overview of Radiomics in Tumor Prognosis Prediction

Radiomics contributes to prognostic prediction in multiple cancer types. MRI-derived radiomics has been suggested to predict breast cancer patients’ response to neoadjuvant chemotherapy and to predict the recurrence in breast cancer [17]. Huang et al. found that combining the radiomic signature could improve the disease-free survival estimation ability in the early-stage non-small-cell lung cancer [18]. A representative study by Wang et al. revealed that multiregional radiomics analysis could refine survival prediction for early-stage pure-solid non-small-cell lung cancer (NSCLC), providing superior risk stratification in a 592-patient cohort [19]. Kickingereder et al. indicated that MRI-based radiomic model had superior performance over standard models for survival prediction and stratification in newly diagnosed glioblastoma [20]. For localized prostate cancer, combining MRI features with clinicopathologic variables through support vector machine could achieve high accuracy (92.2%) in predicting clinical outcomes [21], whereas the radiomic texture features could be utilized as potential predictors of 5-year overall survival in advanced-stage colorectal cancer patients [22].

**Figure 1:**
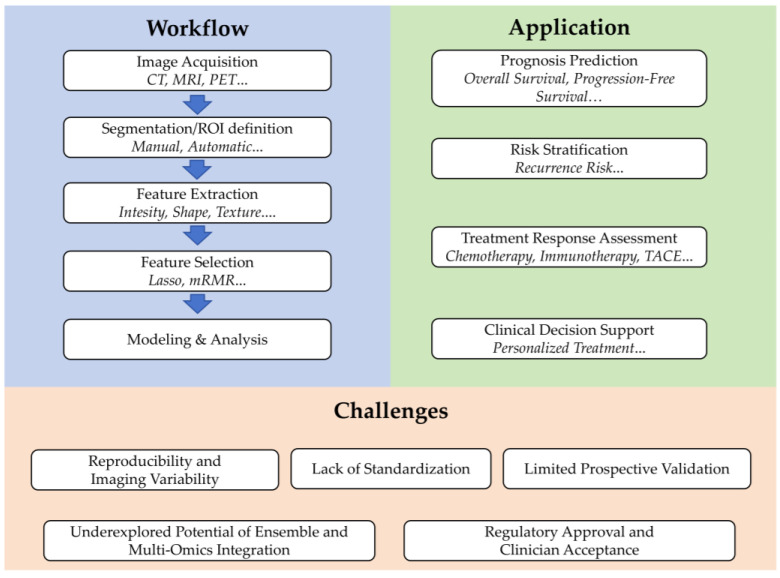
Summary of the features in radiomics-driven tumor prognosis prediction.

**Figure 2:**
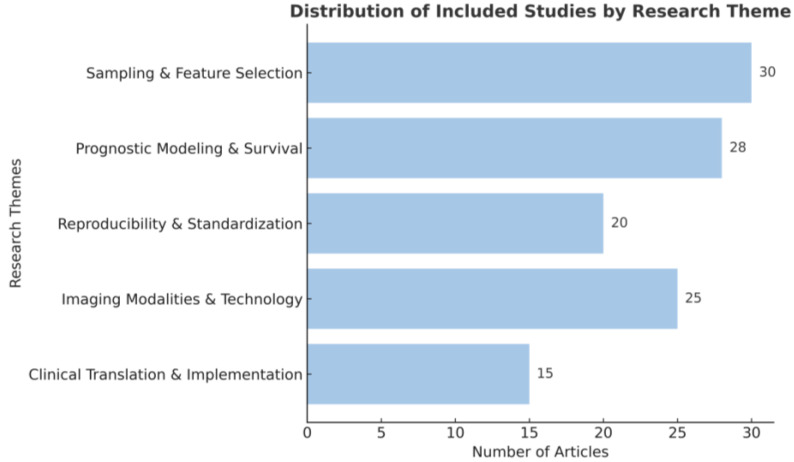
Distribution of the included studies by research theme. The included studies were categorized into five major themes: Sampling & Feature Selection (*n* = 30), Prognostic Modeling & Survival (*n* = 28), Reproducibility & Standardization (*n* = 20), Imaging Modalities & Technology (*n* = 25), and Clinical Translation & Implementation (*n* = 15).

Within the field of radiomics, liver cancer has received comparatively more attention. Kong et al. demonstrated that a radiomics–clinical nomogram could effectively predict treatment response in intermediate–advanced hepatocellular carcinoma (HCC) patients, suggesting its potential utility as an auxiliary diagnostic tool for clinical prognosis assessment [23]. Li et al. developed the radiomics-based method demonstrated superior predictive performance for overall survival and time to progression for patients who treated with apatinib plus trans-arterial chemoembolization (TACE) and this radiomics-driven approach provided a quantitative framework for personalized prognosis prediction and treatment optimization in advanced HCC management [24]. Xu et al. found that pretreatment MRI radiomic features can effectively predict objective response to lenvatinib plus PD-1 inhibitor combination therapy in advanced HCC patients, outperforming conventional clinicopathological factors and significantly correlating with survival outcomes [25]. The overall workflow, clinical applications, and translational challenges of radiomics are summarized in Figure 1. To provide an overview of the literature selection and categorization, the included studies were summarized by major research themes, as illustrated in Figure 2.

## 3. Barriers to Reproducibility and Feature Stability in Radiomics

These presented research results indicated that radiomics is playing an increasingly important role in various aspects of cancer management, including diagnosis, prognosis, and treatment efficacy prediction. However, for a radiomic feature to serve as a reliable biomarker, it must possess the characteristics of high reproducibility. Reproducibility can be assessed in following parts: imaging data reproducibility, segmentation reproducibility, statistical reproducibility, and research reproducibility [26]. Among different categories of features, Zhao et al. found that texture features are unstable and sensitive across reconstruction algorithms, whereas non-texture features (e.g., shape/density features) remain robust [27]. Pfaehler et al. also found textural features are less robust than statistical features and stability was primarily governed by: imaging acquisition, reconstruction settings, segmentation approaches, and interpolation strategies [28]. Shape features were confirmed by Fiset et al. as the most reliable ones of MRI-based radiomic features of cervical tumors [29].

Many factors can affect radiomic features reproducibility, particularly normalization methods [30]. A prostate MRI radiomics study indicated that neither diverse normalization methods nor pre-filtering methods consistently enhanced feature repeatability [31]. Laajili et al. emphasized that feature selection was critical in medical imaging to address high dimensionality by removing irrelevant and redundant features and their results highlighted the challenge of identifying optimal combinations of feature selection techniques and classifiers, noting that morphological features can significantly influence the performance of machine learning algorithms [32]. Demircioğlu et al. identified a challenge in radiomics feature selection, showing that applying selection before cross-validation on high-dimensional datasets can introduce positive bias due to data leakage [33].

## 4. Radiomics Sampling Methods

Radiomics sampling methods mainly focus on specific regions within medical images, such as tumors, lesions, or other structures of interest. Manual segmentation of ROIs remains widely used for feature extraction, but it often suffers from inter-observer variability among radiologists, leading to inconsistencies in model performance [34]. Nevertheless, numerous studies have demonstrated that ROI-based radiomics holds significant prognostic value. The use of automatic or semi-automatic segmentation tools powered by deep learning models like U-Net and no-new-net (nnU-Net) has greatly reduced variation caused by manual operations, enabling more consistent ROI extraction. For example, Park et al. developed a fully automated model based on nnU-Net to segment abdominal solid organs in non-contrast abdominal CT and low-dose chest CT scans. The model achieved Dice similarity coefficients above 0.95 on external test sets, indicating high segmentation accuracy. Moreover, the automatically extracted volume and 3D radiomic features showed strong agreement with those obtained through manual segmentation, suggesting that this approach has promising applications in radiomics analysis [35].

Multi-regional extended ROI analysis includes not only the tumor area but also the peritumoral tissue or even the entire organ. Studies have shown that incorporating peritumoral regions helps capture the tumor microenvironment and improves prediction accuracy. Wang et al. demonstrated that combining radiomic features from both intratumoral and peritumoral regions enhances the prediction of axillary lymph node metastasis in breast cancer patients through a multicenter study. The model performed well by incorporating both tumor and 10 mm peritumoral areas. These findings support the inclusion of peritumoral tissue in ROI analysis to better reflect the tumor microenvironment and improve model performance [36].

Adaptive ROI analysis can dynamically adjust the boundaries of the ROI based on tumor growth patterns or changes observed in imaging over time, which is particularly useful in delta-radiomics. Khorrami et al. analyzed changes in radiomic features from CT scans of NSCLC patients before and after treatment with immune checkpoint inhibitors (delta-radiomics) and found that these changes were closely associated with treatment response and OS. They emphasized that dynamically adjusting the ROI boundaries can help capture changes in the tumor microenvironment and improve the prediction of treatment response [37]. The comparison of recent radiomics sampling methods is summarized in Table 1. These innovative sampling methods not only improve the reproducibility of radiomics research but also enhance its predictive power. However, further standardized validation across more datasets is needed to ensure the general applicability of these methods.

## 5. Recent Advances in Radiomics Feature Selection Methods

Radiomics is a growing field that emphasizes the extraction of high-dimensional, quantitative features from medical images [38]. Overfitting is a common challenge with high-dimensional data [39]. Overfitting happens when a model learns the noise and random variations in the training data instead of the true underlying patterns [40]. Specifically, in a high-dimensional space, it is effortlessly easy to detect spurious, non-generalizable patterns that fit the training data perfectly but fail to accurately predict unseen data. The high-dimensional nature of radiomics renders it particularly susceptible to the “curse of dimensionality,” increasing the risk of model overfitting [41]. Consequently, the risk of model overfitting is arguably the single greatest challenge in translational radiomics. Models developed with overfit features cannot be consistently replicated across different datasets, scanners, or institutions, thereby impeding their clinical implementation.

Therefore, rigorous and deliberate feature selection is not merely an optional optimization step but a critical safeguard against overfitting. It is a necessary process to reduce the dimensionality of the data, isolate the most biologically relevant and stable signals from noise, and ultimately build models that are more likely to generalize. Although, several mistakes can occur in feature selection step, such as cherry-picking features can lead to overfitting of the model [42]. Existing previous research indicates that the model’s performance varies depending on whether feature selection is performed using the entire dataset or solely based on the training and testing sets [43].

The following section reviews feature selection methods, with a critical evaluation of their efficacy in mitigating these specific challenges. Filter-based feature selection methods are essential in radiomics to address challenges associated with high-dimensional data. Techniques such as analysis of variance (ANOVA) and Pearson correlation evaluate the relevance of individual features. The Least Absolute Shrinkage and Selection Operator (LASSO) was the most frequently used technique for feature selection in radiomics studies, followed by Recursive Feature Elimination (RFE) and minimum Redundancy Maximum Relevance (mRMR) [44]. Also, there is challenge in selecting optimal feature selection methods for model performance and stability [44]. LASSO has certain limitations, such as low repeatability and reproducibility of textural features in clinical settings, as well as its restricted ability to effectively model small, unbalanced sample size problems [45]. Meanwhile, mRMR should be carefully used to avoid including redundant variables or excluding clinically relevant ones [45]. Sun et al. found that both feature selection methods and classifiers affect predictive performance in radiomics analysis, offering a strategy for optimizing machine learning models in radiomics-based predictions [46]. Demircioğlu et al. found that simpler methods such as ANOVA, LASSO, and mRMR ensemble demonstrated better predictive performance by testing 29 feature selection methods and 10 classifiers [47].

Recently, a multi-objective based feature selection (MO-FS) algorithm that simultaneously considers sensitivity and specificity for selecting optimal radiomic features has been suggested for good classification performance [48]. Wang et al. proposed a multi-level feature selection algorithm based on Lasso coefficient thresholds (Coe-Thr-Lasso) to address high dimensionality and redundancy in radiomics [49]. The feature subset quality is improved by eliminating features that have weak correlation with classification results. Another team developed a novel feature selection criterion named minimum Redundancy, Maximum Relevance and Maximum Sparse Representation Coefficient (mRMRMSRC) for MRI radiomics, which could produce the area under the curve (AUC) of 90% [50]. In addition, Wilcoxon-based feature selection combined with random forest classification has been suggested to form the most stable and high-performing machine learning pipeline for prognostic modeling, with classifier selection being the predominant factor influencing predictive performance. This enables the development of robust radiomic biomarkers in clinical translation [51]. In general, these approaches enhance model interpretability and computational efficiency but may overlook complex interactions between features. The strengths and weaknesses of the various radiomics feature selection methods are summarized in Table 2.

Recent advancements have integrated these techniques into hybrid frameworks to improve robustness; however, standardization across diverse datasets remains a significant challenge. Meanwhile, the stability and consistency of radiomic features can vary depending on image quality and the software used for feature extraction. Currently, studies on the repeatability and reproducibility of radiomic features are limited to few cancer types [62]. These challenges will be discussed more in the later section.

## 6. Application of Radiomics Across Different Imaging Modalities

Medical imaging plays a vital role in clinical diagnostics and treatment, with CT and MRI being two important modalities. CT offers rapid, high-resolution anatomical visualization with excellent contrast for bones and lung tissue, while MRI provides superior soft tissue contrast and functional insights without the use of ionizing radiation. Due to inherent differences in physics, resolution, contrast mechanisms, and susceptibility to artifacts, CT and MRI exhibit distinct behaviors in image analysis. Recognizing these modality-specific advantages and differences is crucial for optimizing radiomics analysis and ensuring reliable clinical and research outcomes. Understanding the comparative strengths and limitations of CT versus MRI in disease-specific radiomics applications holds critical clinical and research significance.

Meng et al. demonstrated comparable performance between CT-based and MRI-based radiomics models for predicting microvascular invasion in HCC, with MRI showing marginally superior predictive capability and significant added value specifically for 2–5 cm tumors [63]. Another study by Cheng et al. developed and validated CT-based and MRI-based deep learning radiomics models for differentiating intrahepatic cholangiocarcinoma from other primary liver cancers, demonstrating comparable diagnostic performance between modalities with no statistically significant differences [64]. Li et al. also demonstrated comparable performance between CT and multiparametric MRI radiomics for predicting pathological response to neoadjuvant chemotherapy in gastric cancer, while the combined multimodal radiomics nomogram achieved superior predictive accuracy and showed significant prognostic value for overall survival and progression-free survival [65]. The study by Cheng et al. indicated that MRI-based radiomics models outperformed CT-based models in predicting malignant potential of intraductal papillary mucinous neoplasms, while also showing superior feature reproducibility [66]. Liu et al. found that while MRI outperformed CT in evaluating parotid tumor margins, both modalities showed comparable diagnostic accuracy when using radiomics signatures for differentiating pleomorphic adenomas from Warthin tumors [67]. The result of a multicenter study of 234 rectal adenocarcinoma patients indicated that MRI-based radiomics outperformed CT-based models for lymph node metastasis prediction (AUC, 0.785 vs. 0.721, *p* < 0.05) [68]. One prospective study of prostate cancer patients undergoing three-dimensional conformal radiation therapy (3DCRT) demonstrated that MRI-based radiomics combined with clinical information outperformed CT-based approaches in predicting radiation-induced rectal toxicity, with particularly high AUC and specificity (AUC: 0.79, specificity: 82.15%) [69]. In general, both CT-based and MRI-based approaches are widely used though the performance may vary between cancer sites.

Ultrasound, radionuclide imaging, and interventional radiology are not as dominant as CT and MRI in radiomics research, but they have gradually been included in the field. Ultrasound has gained attention in radiomics due to its non-invasive nature, real-time imaging capability, and cost-effectiveness. Studies have shown that texture features extracted from ultrasound images can be used to evaluate breast cancer patients’ response to neoadjuvant chemotherapy. For example, Kim et al. developed and validated a deep learning-based radiomics nomogram using pre- and post-treatment ultrasound to predict pathological complete response (pCR) in patients undergoing neoadjuvant chemotherapy for breast cancer, achieving strong predictive performance (AUC = 0.91–0.97) [6].

Radionuclide imaging, especially PET, is increasingly employed in radiomics research. Liu et al. found that radiomic features extracted from pre-treatment ^18^F-Fluorodeoxyglucose (FDG) PET images could predict survival in patients with locally advanced cervical cancer treated with concurrent chemoradiotherapy. Their study included clinical variables and PET/CT radiomics features, and the combined model predicted 3-year progression-free survival with AUCs ranging from 0.661 to 0.775 across different cohorts [70]. Hyun et al. demonstrated that a PET-based radiomic model, incorporating clinical features and radiomic features, could effectively diagnose lung adenocarcinoma (AUC = 0.859) [71]. Roy et al. optimized robust FDG-PET radiomic signatures using patient-derived tumor xenografts to predict and assess response to therapy in triple-negative breast cancer [72]. Li et al. developed a radiomics nomogram based on ^18^F-FDG PET, achieved an AUC of 0.891 for predicting microvascular invasion and a C-index of 0.831 for disease-free survival in very-early- and early-stage HCC patients, demonstrating a significant improvement over clinical models and potential for personalized risk stratification [73]. Gómez et al. investigated combinations of feature selection methods and machine learning classifiers to predict the metabolic response of metastatic breast cancer lesions using ^18^F-FDG PET/CT radiomic and clinical features [74]. Ni et al. found that combining radiomic features extracted from ^18^F-FDG PET/CT images with clinical data in newly diagnosed multiple myeloma patients yielded higher prognostic performance (C-index 0.790 in training, 0.698 in validation) compared to models using only radiomics or clinical data [75]. However, PET-based radiomics remains somewhat speculative, especially when images are retrospectively collected without access to raw data, limiting the ability to optimize images for radiomics analyses [76]. In contrast, prospectively acquired images with raw data storage provide better opportunities for research and potential improvements, yet the overall reliability and standardization of PET-based radiomics still need further validation.

Radiomics studies in interventional radiology mainly focus on analyzing pre- and post-procedure imaging to assess treatment response and predict prognosis. Deng et al. evaluated the effectiveness of radiomics in predicting outcomes of HCC patients treated with TACE. The results showed that the radiomics model had good predictive power, with an AUC of 0.90 for prognosis following TACE treatment [8]. Zhu et al. developed CT-based radiomics models to predict early efficacy of microwave ablation in malignant lung tumors. Their final model demonstrated clinical benefit in predicting progression-free survival, suggesting utility for individualized risk classification and treatment [77]. Li et al. developed an ultrasound-based radiomics model to predict the volume reduction ratio following microwave ablation in benign breast tumors. Their model performed well by utilizing 846 radiomic features (AUC = 0.93 in validation set, 0.95 in test set) [78]. These studies show that different imaging modalities have strong application potential in radiomics research, especially in tumor characterization, treatment response evaluation, and prognosis prediction.

## 7. Emerging Trends and Techniques

Ensemble feature selection methods have received increasing attention in radiomics. These methods combine multiple feature selection techniques and often perform better than using a single method. They help improve model stability, robustness and generate more reliable feature subsets [79]. For example, Lee et al. used ensemble learning with a single MRI sequence to distinguish between benign and malignant tumors. By preserving information from different MRI sequences, they achieved better prediction performance (AUC = 0.752) [80]. Ensemble methods often use a combination of multiple techniques. This approach takes advantage of the complementarity between individual classifiers to reduce bias and improve model reproducibility. For example, highly relevant features can first be selected using mRMR or ANOVA, and then further reduced with LASSO or Random Forest. This method helps improve model accuracy and interpretability by balancing feature relevance and redundancy [81]. Recent studies have shown that ensemble feature selection can improve model reproducibility and generalization by controlling overfitting and adjusting for multiple comparisons [82]. In radiomics, the number of features is usually much greater than the number of samples, these methods can help reduce the risk of overfitting especially when dealing with data heterogeneity or multi-center studies [26].

Another emerging research trend is the integration of radiomics with multi-omics data, such as genomics, transcriptomics, and proteomics, to gain a more comprehensive understanding of biological systems. Radiomics can be combined with genetic data to build connections between genomic information and imaging features, helping to explore the underlying biological mechanisms of tumors. This strategy can capture the molecular phenotype of cancer and has great potential to improve cancer prediction and diagnosis [16]. For example, Wang et al. extracted and selected radiomic features to analyze the relationship between tumor phenotypes in CT images and gene expression among 89 NSCLC patients. The results showed that the radiomics model predicted metagenes with an accuracy ranging from 41.89% to 89.93%. This successfully reflected important biological information in NSCLC patients and could improve clinical decision-making at a low cost [83]. In glioblastoma research, Luan et al. confirmed that radiomic features based on transcriptomic data and MRI could divide patients into high-risk and low-risk groups. The clinical model showed an AUC of 0.83, and its ability to stratify patient risk demonstrated great potential for clinical translation [84].

Currently, the integration of artificial intelligence (AI)-driven multi-omics and radiomics is a leading area of research. By combining biological data with radiomic features and using AI techniques, this approach has shown great potential in improving cancer diagnosis, classification, and prognosis [16]. For example, Chen et al. showed that using AI to combining radiomic features and genomic data significantly improved the performance of overall survival prediction models in NSCLC patients (C-index = 0.85) [85]. Researchers have been exploring advanced AI-based sampling techniques and feature selection methods to further improve the reliability of models. Bernatowicz et al. explained intratumoral and intertumoral heterogeneity by calculating voxel-based radiomic features [86]. Voxel size resampling is a key step in radiomics image preprocessing and has an important impact on radiomic features [87]. These studies suggest that integrating radiomics with multi-omics data not only improves prediction accuracy but also provides important insights into tumor biology.

## 8. Clinical Translation

Translating radiomics-based models from research into clinical practice is gradually becoming a new focus. Traditional medical imaging plays an important role in diagnosis, and with the development of radiomics, it can greatly reduce misdiagnosis and missed diagnoses [88]. Radiomics, powered by AI algorithms, has significantly improved the performance of sampling techniques and feature selection models in clinical translations such as predicting cancer metastasis, recurrence, treatment response, and complications [13]. Current emerging technologies focus on computational efficiency, interpretability, and real-time analysis. This includes developing lightweight algorithms that reduce the need for heavy computing resources, making it possible to use these models in hospital systems with limited infrastructure. For example, Mostafa et al. found that the performance of radiomics prediction models can be improved by using a reduced feature set. After applying this technique, the accuracies of HCC prediction models using K-nearest neighbor (KNN), decision tree, neural network, and support vector machine (SVM) algorithms are very high (AUC = 94–97%) [89]. Bibault et al. used a radiomics model based on a deep neural network classifier combined with clinical data to predict post-radiotherapy rectal cancer patients’ treatment response with 80% accuracy. For these patients, long-term conservative treatment with fewer side effects could be used. The model also achieved a short processing time [90]. In a lung cancer study, Zhang et al. developed a deep learning model based on a 3D ResNet architecture to predict the risk of radiation pneumonitis by combining CT images with radiation dose maps. The research team also created an online software tool to support the practical use in clinical settings [91].

Compared to feature extraction and modeling, sampling methods such as organ contouring are also key to improving model performance. However, due to the complexity of human body, different doctors may have different opinions on the lesion, leading to low consistency in contouring. Some researchers have tried to include certain radiomic features to improve the accuracy of segmentation. Torrents-Barrena et al. first extracted radiomic features to describe the anatomical structures in MRI. They used K-best and forward feature selection to identify the best radiomic features for each structure and then combined these features with SVM to achieve accurate segmentation [92]. Park et al. used the nnU-Net model to achieve accurate segmentation of solid organs in non-contrast abdominal and low-dose chest CT scans, which allowed for stable extraction of radiomic features [26].

The clinical significance of radiomics lies in its potential to revolutionize oncology care through non-invasive, data-driven biomarkers. Ren et al. developed a CT-based radiomics model which holds promise as a non-invasive approach for distinguishing between early and late stages of pancreatic ductal adenocarcinoma [93]. The radiomics-based machine learning model developed by Li et al. can enhance the prediction of clinically significant prostate cancer, demonstrating improvements in both diagnostic accuracy and overall clinical benefit [94]. In clinical practice, efficient prediction of treatment outcomes in cancer patients using radiomics is warranted. Yan et al. used radiomic feature analysis from CT images to predict the efficacy of radiotherapy in lung cancer patients. They found that flatness and coefficient of variation had good predictive performance when using SVM and suggested that low-order features (such as shape and intensity) performed better than high-order features [95]. In addition, a study on primary nasopharyngeal carcinoma built radiomics models based on pre-treatment MRI and delta radiomics features before and after induction chemotherapy (IC) to predict the effectiveness of IC combined with concurrent chemoradiotherapy among 272 patients. Features were selected using mRMR and LASSO, and the models achieved AUCs of 0.865 and 0.941, respectively. These results suggest that the delta model has potential value in predicting treatment response [96].

Radiomics has also shown great potential in the early assessment of toxicity. Modern radiotherapy techniques inevitably expose nearby organs around the target area to radiation. Short-term toxicity usually appears during or within 3 months after radiotherapy and often resolves shortly. However, some side effects are irreversible and develop over time, such as tissue fibrosis and radiation pneumonitis. Therefore, timely toxicity assessment is very important [97]. In predicting radiotherapy toxicity for lung cancer, traditional models based on clinical data and dose–volume histograms have low accuracy for clinical use. Bourbonne et al. included 167 lung cancer patients and found that radiomic features based on 3D dose maps significantly improved prediction performance. The combined model achieved a balanced accuracy of 0.92 for acute lung toxicity and 0.89 for late lung toxicity, which was much better than the traditional models [98].

To conclude, quantitative imaging techniques, combing radiomics and deep learning, are ideal methods for personalized patient management. It can offer highly accurate and reproducible tools that help reduce costs and save time [99]. Future improvements to radiomic models such as incorporating genomics and histology data, have the potential to enhance prediction accuracy and model robustness, aligning with the broader advancements in multimodal artificial intelligence in oncology [100].

## 9. Challenges and Future Directions

Although the reviewed studies show that radiomics has made significant progress in cancer research in recent years, there are still some limitations that need to be addressed before it can be widely used in clinical practice. These limitations involve technical, methodological, and clinical aspects. Solving them is essential for improving model performance, reliability, and clinical relevance.

### 9.1. Reproducibility and Imaging Variability

Reproducibility is one of the most urgent issues that needs to be addressed in radiomics research. This issue mainly involves ROI segmentation and detail consistency. As different doctors may define tumor boundaries in different ways, manual segmentation often has low reproducibility. Segmentation variability, especially manual tumor contouring, introduces inconsistency that affects the extracted features and reduces the robustness of the model. Advanced automatic segmentation tools and atlas-based contouring methods have shown potential, but they still need further validation in different clinical settings [101]. Moreover, existing deep learning models perform well only on specific datasets, the lack of standardization in the formatting of databases impedes the ability to integrate multiple data sources within the same machine learning algorithm [13,102]. Radiomic features are highly sensitive to changes in imaging protocols, scanner types, reconstruction algorithms, preprocessing steps, and patient populations across different platforms [16]. Existing radiomics guidelines may not fully accommodate the wide variety of scanners, protocols, and reconstruction parameters employed by different vendors across multiple centers [103]. Studies have shown that even small changes in parameters such as slice thickness, field of view, or voxel size can cause significant differences in extracted features. One study found that slice thickness in CT scans had a strong impact on radiomic features in lung tumors. Among different features, shape and first-order features were more reproducible, while texture features had lower reproducibility [104]. These differences make it difficult to reproduce results across datasets and reduce the generalizability of radiomics models. Although the Image Biomarker Standardization Initiative (IBSI) has worked to standardize the radiomics workflow, it cannot ensure that all software used in studies follows IBSI reference standards [105]. Differences in software, design choices, or parameter settings still lead to limited consistency across studies.

### 9.2. Lack of Standardization in Feature Selection Methods

Feature selection plays a vital role in reducing overfitting and improving the interpretability of radiomics models. However, there is currently no consensus on the best approach to select features from the high-dimensional radiomic datasets. Studies use a wide range of methods—such as ANOVA, mRMR, LASSO, and RFE—but often fail to justify their choices or provide rationale for parameter tuning. As a result, the same data processed with different methods may lead to different feature sets and inconsistent outcomes. For example, Hua et al. used seven types of classifiers (such as SVM and linear discriminant analysis) along with different types of feature distributions (linear, non-linear, and bimodal Gaussian models) to explore the relationship between the number of features and the size of the training dataset. Using real breast cancer patient data, they found that different models selected different numbers of features, which largely depended on the type of classifier and the distribution of feature [106].

In addition, some studies perform feature selection before cross-validation, which leads to data leakage and overly optimistic performance. These methodological issues reduce the reliability of the model results and limit their reproducibility. Demircioğlu et al. conducted a study using 10 publicly available radiomics datasets, where feature selection was applied before cross-validation. The results showed that using the wrong order of feature selection could lead to an AUC-ROC bias of up to 0.15, an AUC-F1 bias of up to 0.29, and an accuracy bias of up to 0.17 [33]. Therefore, developing a standardized and transparent feature selection process, including preprocessing, resampling, and validation, would greatly support the progress of radiomics research.

### 9.3. Limited Prospective Validation in Clinical Settings

Another major challenge is the lack of large-scale prospective validation, and poor model generalization prevents wide clinical translation [107]. At present, there is no universal method for all scenarios. Simply extracting radiomic features in a robust way is not enough to improve model generalizability. Most published studies are based on retrospective analyses from single-center cohorts. Oliveira et al. pointed out that radiomics studies should use multi-center imaging data to support external validation. They found that prediction models built on standardized multi-center datasets showed good performance, with an AUC as high as 0.74 in the validation cohort for predicting event-free survival in NSCLC patients [108]. Although standardized multi-center studies help increase the potential for clinical translation, challenges such as the lack of real-time evaluation and limited sample diversity remain. More prospective, multi-center studies are essential to test the clinical usefulness of radiomics models.

In addition, integration into hospital workflows also requires a combination of efforts, including clinical impact assessment, cost-effectiveness analysis, and proof of improved patient outcomes. A systematic review of radiomics in neuro-oncology pointed out that cost-effectiveness studies are needed after biological and clinical validation. Such studies help ensure that radiomics provides better value compared to existing biomarkers. This research also noted that the lack of prospective studies and external validation limits the clinical usefulness of radiomics, and that further technical and clinical validation strategies are still needed to support clinical integration [109]. In conclusion, radiomics models need to be effectively integrated into hospital workflows and undergo comprehensive validation. This includes structured evaluation frameworks and prospective studies, which are key to promoting the clinical translation of radiomics.

### 9.4. Underexplored Potential of Ensemble and Multi-Omics Integration

Ensemble feature selection and multi-omics integration are powerful tools in radiomics that have not yet been fully explored. Ensemble methods improve model stability and robustness by combining the results of different feature selection techniques and reducing reliance on a single method. Similarly, integrating radiomics with genomics, transcriptomics, or proteomics can enhance prediction performance and provide biological interpretability related to tumors. However, research on these strategies is still in the early stages. Several challenges must be addressed when performing such data integration, including increased data dimensionality, higher computational costs, and the coordination across different types of datasets. Some studies have pointed out that multi-omics datasets often have high dimensionality and sparsity, which can make it difficult for traditional statistical methods to handle the “curse of dimensionality” [110]. To address these challenges, researchers have developed various data integration methods, including autoencoder based approaches for multi-omics data integration in cancer survival prediction [111,112,113,114]. Although these methods have shown potential in theory and experiments, practical applications still face issues such as data standardization, consistency in feature selection, and model interpretability. Future research should focus on developing efficient, scalable, and interpretable integration methods to support the wider clinical use of radiomics.

### 9.5. Regulatory Approval and Clinician Acceptance

While radiomics and AI show immense diagnostic and prognostic potential, their translation into routine clinical practice faces significant challenges, primarily concerning regulatory approval and clinician acceptance. Bera et al. indicated that the regulatory approval pathway is a major obstacle to the clinical adoption of imaging-based AI prognostic and predictive tools, with one key requirement being the clear explanation of the software functions [115]. Funingana et al. suggested that creating strategies to standardize segmentation, validation, and data sharing could accelerate the regulatory approval process and facilitate the adoption of new radiomic biomarkers [116]. Despite numerous studies suggesting the value of radiomics for diagnosis and monitoring of diseases, its limited routine use in clinical trials is primarily due to lack of standardization, insufficient validation in large multicenter trials, limited focus on interpretability and biological relevance, and poorly defined criteria for assessing study quality [117]. Meanwhile, Pesapane indicated that the ethical imperative for stringent regulation of AI in radiomics is underscored by the potential for misuse driven by profit motives, which may prioritize financial gain over patient care in clinical decisions [118].

## 10. Conclusions

In this review, we have synthesized the notable advancements in radiomics sampling methodologies and feature selection algorithms in a range of imaging modalities. The evolution from traditional region-based sampling strategies to voxel-level analytical techniques and deep learning-based feature extraction has significantly enhanced the accuracy and clinical applicability of radiomics predictive models. Furthermore, the adoption of ensemble feature selection approaches and the integration of multi-omics data have substantially improved model robustness and biological interpretability.

Despite these advancements, the translation of radiomics into clinical practice remains challenging. Key obstacles include limited reproducibility due to variations in imaging protocols, inconsistencies in manual segmentation, and a lack of standardization in feature selection. Many studies continue to rely on retrospective single-center data, underscoring the need for large-scale prospective validations and clinically adaptable standardized workflows. Moreover, although ensemble learning and multi-omics integration hold considerable promise, their widespread implementation is hindered by high computational demands, data complexity, and scarce availability of harmonized multi-modal datasets. Additionally, the pathway to regulatory approval and broader clinician acceptance remains challenging. Robust technical validation and clear demonstrations of clinical utility and interpretability that align with regulatory standards are needed for incorporation into existing clinical workflows.

Future work should focus on developing standardized workflows for radiomics sampling and feature selection, strengthening multi-center collaboration, and conducting large-scale prospective clinical studies. Technological innovations will help accelerate the clinical use of radiomics, such as interpretable AI models, lightweight algorithms suitable for real-time use, and efficient multi-omics integration frameworks. By addressing these challenges and building on current progress, radiomics has the potential to become an indispensable tool in tumor risk stratification, personalized treatment planning, and prognosis prediction.

## Figures and Tables

**Table 1:** Comparison of Radiomics Sampling Methods.

Sampling Method	Key Features	Benefits	Limitations
Manual ROI Segmentation [34]	Widely used; prone to inter-observer variability	Simple and interpretable	Observer-dependent; inconsistent
Automatic/Deep Learning-Based Segmentation [35]	Uses models like U-Net/nnU-Net; reduces variability; consistent results	High accuracy; reproducibility	Requires computational resources and model training
Multi-Regional Extended ROI [36]	Includes tumor and peritumoral regions; captures microenvironment	Improved prediction accuracy; reflects tumor microenvironment	Complex ROI definition; more data required
Adaptive ROI (Delta-Radiomics) [37]	Dynamically adjusts ROI based on tumor evolution; supports delta-radiomics	Captures temporal changes; enhances prediction of treatment response	Needs longitudinal data; complex implementation

**Table 2:** Comparison of Radiomics Feature Selection Methods.

Method	Benefits	Limitations
ANOVA	Simple, interpretable; good for identifying statistically significant features [52]	Ignores feature interdependencies [53]
Pearson Correlation	Easy to compute; identifies linear relationships [54]	Only captures linear relationships; may miss nonlinear patterns [54]
Least Absolute Shrinkage and Selection Operator (LASSO)	Performs both variable selection and regularization; enhances generalization [55]	Exclude weakly informative features [56]
Recursive Feature Elimination (RFE)	Systematic backward feature elimination; effective in small feature sets [57]	Computationally expensive for large datasets [58]
Minimum Redundancy Maximum Relevance (mRMR)	Balances relevance and redundancy; commonly used in radiomics [59]	Time-consuming; insufficient for coping with high-dimensional pattern classification [60]
Multi-objective Feature Selection (MO-FS)	Considers multiple objectives (sensitivity, specificity); promising classification	May require large datasets; balancing objectives can be complex [61]
Lasso coefficient thresholds (Coe-Thr-Lasso)	Reduces dimensionality and redundancy; enhances subset quality	Threshold selection sensitive; model-specific tuning needed [49]
Minimum Redundancy, Maximum Relevance and Maximum Sparse Representation Coefficient (mRMRMSRC)	Improves sparse representation and relevance; high AUC performance	High model complexity; computational burden [50]
Wilcoxon + Random Forest	Stable and high-performing; good for prognostic modeling	Classifier-dependent; may not generalize across all datasets [51]

## Abbreviations

The following abbreviations are used in this manuscript:

CT	Computed tomography
MRI	Magnetic resonance imaging
PET	Positron emission tomography
ROI	Region of interest
ANOVA	Analysis of variance
LASSO	Least absolute shrinkage and selection operator
mRMR	Minimum redundancy maximum relevance
NSCLC	Non-small-cell lung cancer
HCC	Hepatocellular carcinoma
TACE	Transarterial chemoembolization
nnU-Net	No-new-net
RFE	Recursive Feature Elimination
mRMRMSRC	Minimum Redundancy, Maximum Relevance and Maximum Sparse Representation
AUC	Area under the curve
MO-FS	Multi-objective Feature Selection
Coe-Thr-Lasso	Lasso coefficient thresholds
3DCRT	Three-dimensional conformal radiation therapy
pCR	Pathological complete response
FDG	Fluorodeoxyglucose
AI	Artificial intelligence
KNN	K-nearest neighbor
SVM	Support vector machine
IC	Induction chemotherapy
IBSI	Image Biomarker Standardization Initiative
